# Lectures on the Physiology of the Circulation

**Published:** 1859-11

**Authors:** 


*Lectures on the Physiology of the Circulation.*—We commence with this number, a series of lectures on the above subject, by Prof. Dalton, of the College of Physicians and Surgeons. This course consists of sixteen lectures, which will appear monthly from this time. The preliminary term of our colleges affords an excellent opportunity for the elaboration of special subjects; and the course which we have now commenced to publish will be complete in every respect. The established reputation of Dr. Dalton is a guarantee of the interest and importance of these lectures, which will contain much new and entirely original matter, elucidating many points in regard to the circulation which have heretofore been involved in obscurity. Published, as they will be, under the personal supervision of the author, they will appear in the best possible form, elaborated rather than otherwise, from the lectures as actually delivered. We conceive that more interesting matter has never been presented to the readers of our Journal; and are gratified, so soon after our removal to New York, to receive such valuable assistance from one who has taken the pre-eminent stand in his department in this country.

